# The impact of liver transection depth on surgical difficulty in robotic versus laparoscopic limited liver resection (TAKUMI-5)

**DOI:** 10.1007/s00423-025-03916-0

**Published:** 2025-11-27

**Authors:** Tomokazu Fuji, Kosei Takagi, Kazuya Yasui, Atene Ito, Takeyoshi Nishiyama, Yasuo Nagai, Shohei Yokoyama, Toshiyoshi Fujiwara

**Affiliations:** Department of Gastroenterological Surgery, Dentistry, and Pharmaceutical Sciences, Okayama University Graduate School of Medicine, 2-5-1 Shikata- cho, Kita-ku, Okayama, 700-8558 Japan

**Keywords:** Robotic surgery, Laparoscopic surgery, Limited liver resection, Textbook outcome

## Abstract

**Purpose:**

Although robotic liver resection (RLR) has gained popularity worldwide, limited liver resection remains the mainstay of RLR. This study aimed to investigate the effect of parameters, including liver transection depth (LTD), on surgical difficulty in limited RLR compared with limited laparoscopic liver resection (LLR).

**Methods:**

This retrospective study included 105 patients who underwent limited RLR (*n* = 56) or LLR (*n* = 49) at our institution between January 2018 and December 2024. After comparing outcomes of RLR and LLR, multivariate analyses were performed to examine effect of LTD on surgical difficulty (defined as prolonged operative time). Moreover, outcomes stratified by LTD cut-off values were compared between the groups.

**Results:**

Median LTD was similar between groups (RLR vs. LLR: 2.6 vs. 2.6 cm, *P* = 0.77). LTD was significantly correlated with operative time for both procedures (RLR, R² = 0.07, *P* = 0.042; LLR, R² = 0.08, *P* = 0.046). Multivariate analyses demonstrated that LLR (odds ratio, 6.9; *P* < 0.001) and LTD (odds ratio, 2.0; *P* = 0.004) were significant risk factors of surgical difficulty. Among patients with deeper LTD (> 2.5 cm), the RLR group had significantly shorter operative time (145 vs. 231 min, *P* < 0.001), less blood loss (nil vs. 100 mL, *P* = 0.006), and a higher rate of textbook outcomes (76.7% vs. 42.3%, *P* = 0.01).

**Conclusion:**

This study investigated impact of LTD on surgical outcomes in patients who underwent limited RLR compared to those who underwent limited LLR. LTD may be a useful parameter for estimating surgical difficulty in limited RLR. Moreover, robotic surgery may be favorable for deeper and limited liver resections.

**Supplementary Information:**

The online version contains supplementary material available at 10.1007/s00423-025-03916-0.

## Introduction

Robotic liver resection (RLR) has become increasingly popular worldwide, supported by growing evidence [1]. RLR is currently indicated for various types of hepatectomies in high-volume centers, and the potential advantages of RLR over laparoscopic liver resection (LLR) have been reported [2, 3]. However, limited liver resection remains the mainstay of RLR at many centers. Therefore, determining the surgical indications for limited RLR to maximize its benefits over limited LLR remains controversial.

To date, several scoring systems have been developed to evaluate the surgical difficulty of LLR [4, 5]. However, few studies have investigated the impact of tumor characteristics on surgical difficulty in RLR. As surgical difficulty may vary depending on the tumor depth and diameter, even with limited liver resection [6, 7], we hypothesized that liver transection depth (LTD) influences the surgical difficulty of limited RLR.

This study aimed to investigate the impact of LTD on the outcomes of patients who underwent limited RLR compared with those who underwent limited LLR. Moreover, we evaluated the significance of robotic surgery in challenging cases such as deeper limited liver resection. This study was part of a training program for minimally invasive surgery (TAKUMI-5) at Okayama University.

## Materials and methods

### Study design

This single-center retrospective study included 249 consecutive patients who underwent RLR or LLR at our hospital between January 2018 and December 2024. To evaluate the effect of LTD on outcomes, we included patients who underwent limited RLR or LLR for a single lesion. This study was approved by the Ethics Committee of our institution (approval number 2503-004) and was conducted in accordance with the principles of the Declaration of Helsinki (2024). The requirement for informed consent was waived due to the retrospective design of the study. This study was in line with the STROCSS 2024 guideline [8].

### Definition of limited liver resection

Regarding the extent of liver resection, partial resection of any segment is considered limited [5]. Patients who underwent left lateral sectionectomy, segmentectomy, or sectionectomy or more were excluded.

### Data collection

Using a prospectively collected database, we extracted the following data: age, sex, body mass index, Child-Pugh classification, primary disease (including hepatocellular carcinoma and metastatic tumor), history of abdominal surgery, tumor factors (size and location), surgical difficulty level (evaluated using the Iwate Criteria) [5], operative factors (type of resection [cutting or scooping], operative time, estimated blood loss, conversion to open surgery, liver transection time, and transection speed), pathological factors (margin status and LTD), and postoperative outcomes (mortality, major complications defined as Clavien–Dindo grade ≥ 3a [9], bile leak ≥ grade B [10], hospital stay, readmission within 30 postoperative days, and textbook outcome).

The liver transection time was measured using intraoperative videos from the initial incision of the liver surface until the completion of transection. When the Pringle maneuver was used, the total time included all intermittent clamping periods, but excluded the time for hemostasis after specimen removal. The liver transection speed was calculated by dividing the transection area by the transection time.

A textbook outcome was defined as the absence of specific adverse events, including conversion to open surgery, postoperative bile leak, major complications, readmission within 30 postoperative days, in-hospital mortality, incomplete resection, and prolonged postoperative hospital stay (≥ 75th percentile) [11].

### LTD measurement

**Fig. 1:**
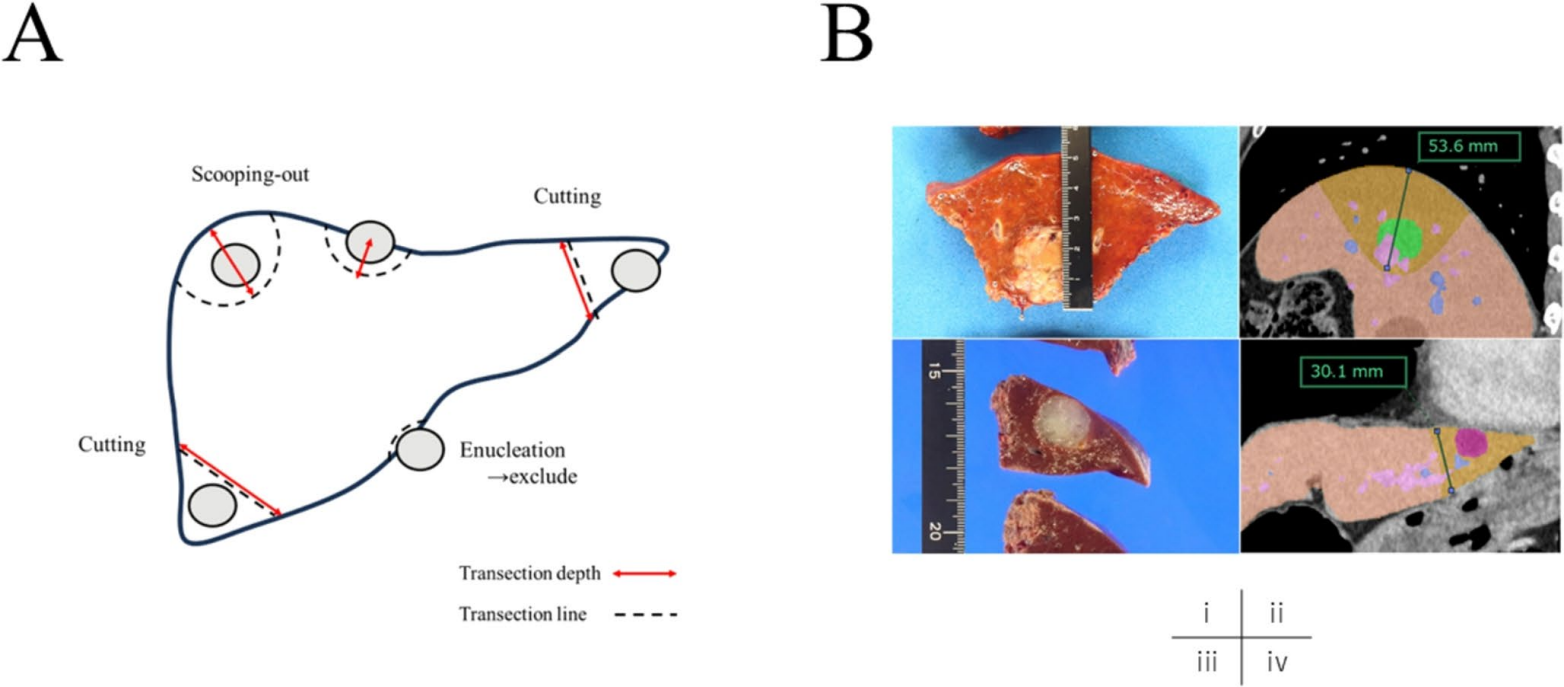
Measurement of liver transection depth. (**a**) Schema of resection types and liver transection depth measurement; (**b**) Measurement of liver transection depth on the specimen and on VINCENT simulation for (i and ii) scooping-out type and (iii and iv) cutting type resections

Transection area simulation was performed using SYNAPSE VINCENT (Fujifilm, Tokyo, Japan) as previously reported [12, 13]. Briefly, the appropriate transection plane was simulated based on a video image showing the surgical field after the completion of liver resection, the weight of the resected specimen, and the specimen shape. LTD was measured using the specimen as the distance from the liver surface to the deepest point for scooping-out resections and as the shortest diameter of the transection plane for cutting resections (Fig. 1). For reference, the transection depth was calculated using the VINCENT simulation and verified by comparison with the actual depth of the specimen.

### Surgical protocol

The details of our RLR protocol using a two-surgeon technique have been reported previously [14].A robotic platform with a da Vinci Si or Xi system (Intuitive Surgical, Sunnyvale, CA, USA) was used. Liver parenchymal transection was performed using the clamp-crush technique by a console surgeon and/or a laparoscopic Cavitron Ultrasonic Surgical Aspirator (CUSA; Integra Lifesciences, Princeton, NJ, USA) or a water-jet scalpel (ERBEJET2; ERBE Elektromedizin, Tübingen, Germany). The Pringle maneuver is typically performed using a tourniquet system. In LLR, the liver parenchyma was transected using CUSA and ultrasonic shear as previously described [12].

### Statistical analysis

Initially, the outcomes of limited RLR were analyzed and compared with those of LLR alone. The association between LTD and surgical parameters, including operative time, transection time, and transection speed, was investigated using linear regression analysis. Subsequently, univariate and multivariate logistic regression analyses were performed to identify the predictors associated with surgical difficulty (prolonged operative time >170 min). In this study, an operative time longer than the median operative time was defined as prolonged operative time based on previous literature [12]. Variables with *P* < 0.01 in the univariate analysis were included in the multivariate analyses. Following evaluation of the cutoff value of LTD by measuring the area under the curve using the receiver operating characteristic curve, outcomes stratified by the cutoff value of LTD were compared between limited RLR and LLR as a subgroup analysis.

Clinical variables were compared using the Mann–Whitney *U* test for continuous data and the chi-squared test or Fisher’s exact test for categorical data. Continuous variables were presented as medians and interquartile ranges (IQRs). In the logistic regression analyses, odds ratios (ORs) and 95% confidence intervals (CIs) were determined. Statistical significance was set at *P* < 0.05. All statistical analyses were performed using JMP, version 14 (SAS Institute Inc., Cary, NC, USA).

## Results

### Study cohort

**Fig. 2:**
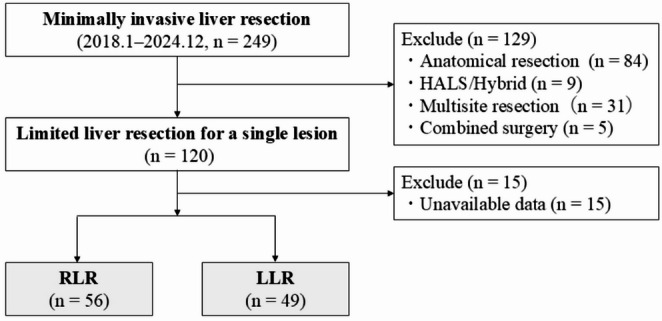
Inclusion flowchart of this study. RLR, robotic liver resection; LLR, laparoscopic liver resection

A flow diagram of this study is shown in Fig. 2. Of 249 patients who underwent minimally invasive liver resection at our institution, 120 who underwent limited liver resection for a single lesion were enrolled. Finally, 56 and 49 patients who underwent limited RLR and limited LLR, respectively, were included in the study.

Characteristics of the 56 patients who underwent limited RLR are shown in Table 1. There were 42 men and 14 women with a median age of 68 years (IQR, 61–76 years). The median operative time was 139 min (IQR, 115–178 min) and the estimated blood loss was nil (IQR, 0–50 mL). The median LTD was 2.6 cm (IQR, 2.0–3.5 cm). The achievement rate of textbook outcomes was 78.6% (*N* = 44).

**Table 1:** Outcomes of robotic and laparoscopic limited liver resection

	RLR (*n* = 56)	LLR (*n* = 49)	*P* value
Patient factor			
Age, years	68 (61–76)	70 (61–74)	0.92
Sex			
Female	14 (25.0)	19 (38.8)	0.13
Male	42 (75.0)	30 (61.2)	
Body mass index, kg/m^2^	23 (21–27)	23 (20–28)	0.79
Child Pugh score A	56 (100)	49 (100)	1.00
Primary disease			
HCC	27 (48.2)	21 (42.9)	0.30
Metastatic tumor	28 (50.0)	24 (49.0)	
Others	1 (1.8)	4 (8.2)	
Previous surgical history			
Upper abdominal operation	19 (33.9)	23 (46.9)	0.18
Hepatectomy	10 (17.9)	11 (22.5)	0.56
Tumor factor			
Tumor size, mm	19 (15–31)	15 (10–25)	0.15
Tumor location			
Anterolateral tumors (S1–6)	40 (71.4)	38 (77.6)	0.48
Posterosuperior tumors (S7 and 8)	16 (28.6)	11 (22.5)	
IWATE Difficulty Criteria			
Low	33 (58.9)	33 (67.4)	0.37
Intermediate	23 (41.1)	16 (32.7)	
Liver parenchymal transection			
CUSA	27 (48.2)	49 (100)	< 0.001
Water jet	5 (8.9)	0 (0)	
Type of resection			
Cutting	21 (37.5)	25 (51.0)	0.16
Scooping out	35 (62.5)	24 (49.0)	
Operation time, min	139 (115–178)	195 (157–245)	< 0.001
Blood loss, ml	0 (0–50)	50 (0–173)	0.002
Conversion to open surgery	0 (0)	4 (8.2)	0.03
Specimen weight, g	39 (20–75)	50 (25–110)	0.15
Positive surgical margin	2 (3.7)	3 (6.4)	0.54
Liver transection depth, cm	2.6 (2.0–3.5)	2.6 (2.1–3.2)	0.77
Liver transection time, min	39 (22–61)	77 (52–102)	< 0.001
Liver transection speed, cm^2^/min	1.00 (0.70–1.48)	0.50 (0.35–0.74)	< 0.001
Postoperative factor			
Mortality	0 (0)	0 (0)	1.00
Major complication (CDc ≥ IIIa)	4 (7.1)	2 (4.1)	0.50
Bile leak ≥ Grade B)	2 (3.6)	0 (0)	0.18
Hospital stay, day	7 (6–8)	7 (6–10)	0.17
Readmission	0 (0)	1 (2.0)	0.28
Achievement of textbook outcome	44 (78.6)	31 (63.3)	0.08

Values are reported as *n* (%), or median (interquartile range)

*RLR* robotic liver resection, *LLR* laparoscopic liver resection, *HCC* hepatocellular carcinoma, *CUSA* Cavitron ultrasonic surgical aspirator, *CDc* Clavien–Dindo classification

### Comparison between limited RLR and LLR

Outcomes of the limited RLR and LLR groups are shown in Table 1. There were no significant differences between the groups in terms of preoperative and intraoperative factors, including tumor characteristics, surgical difficulty level evaluated using the Iwate Criteria, or type of resection. The RLR group had better operative outcomes, with a significantly shorter operative time (139 vs. 195 min, *P* < 0.001), lesser blood loss (nil vs. 50 mL, *P* = 0.002), and a lower conversion rate (nil vs. 8.2%, *P* = 0.03). Liver transection time and transection speed were significantly higher in the RLR group. In contrast, postoperative outcomes were comparable between the groups.

### Association of LTD with surgical parameters between limited RLR and LLR

**Fig. 3:**
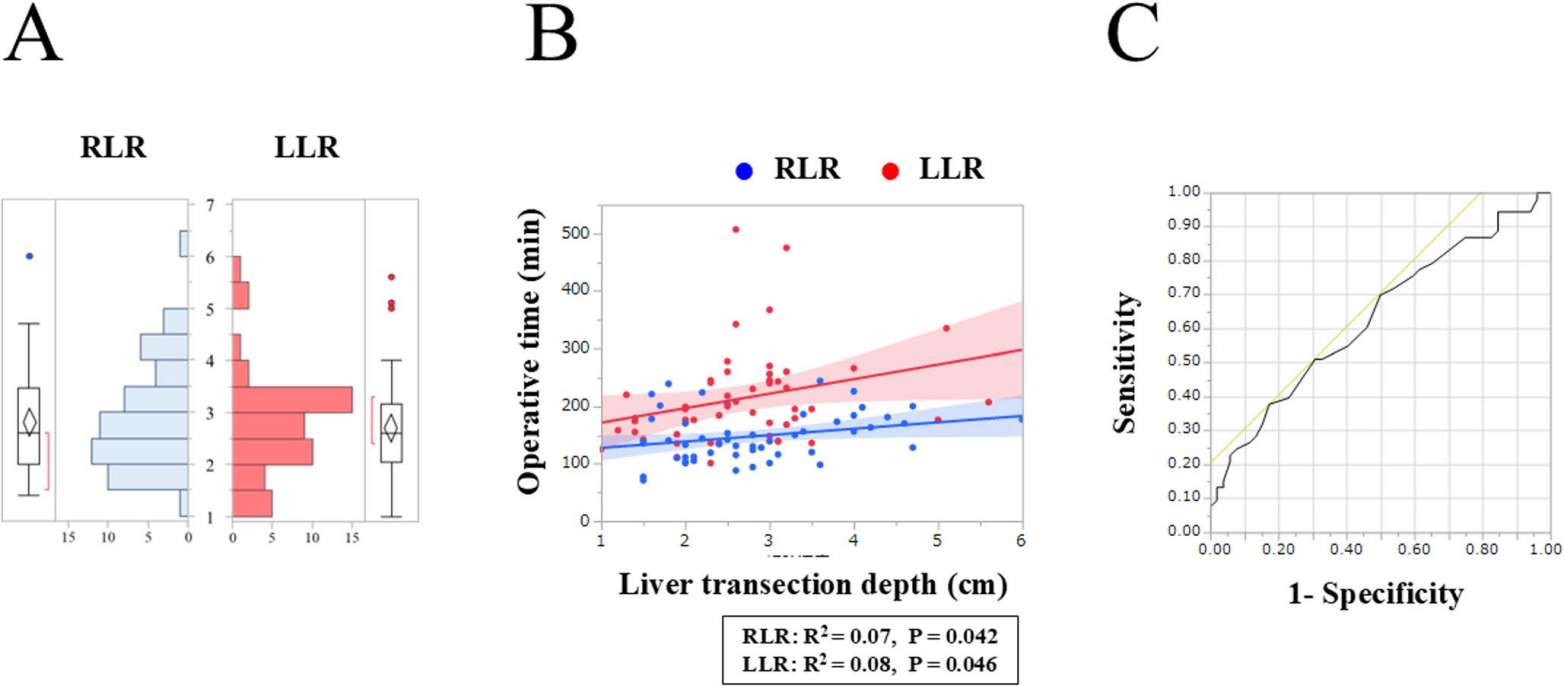
(**a**) Diagram of liver transection depth in limited robotic (RLR) and laparoscopic (LLR) liver resection; (**b**) Relation between liver transection depth and operative time; (**c**) Receiver operating characteristic curve of liver transection depth for prolonged operative time (>170 min), showing the cutoff value of 2.5 cm (area under the curve, 0.63)

The median LTD was similar between groups (RLR vs. LLR: 2.6 vs. 2.6 cm, *P* = 0.77; Fig. 3a). Figure 3b shows a significant positive correlation between LTD and operative time for RLR (R² = 0.07, *P* = 0.042) and LLR (R² = 0.08, *P* = 0.046). There was a strong correlation between LTD and transection time (RLR: R² = 0.26, *P* < 0.001; LLR: R² = 0.25, *P* < 0.001) (Fig. S1a). No correlation was found between LTD and transection speed (RLR, R² = 0.06, *P* = 0.08; LLR, R² = 0.001, *P* = 0.84), whereas the RLR group had a shorter transection time than the LLR group at any transection depth (Fig. S1b).

### Predictive factor associated with surgical difficulty in minimally invasive limited liver resection

The results of the univariate and multivariate analyses investigating the predictive factors related to surgical difficulty (prolonged operative time > 170 min) are shown in Table 2. Multivariate analyses revealed that LTD (odds ratio [OR], 2.0; *P* = 0.004) and LLR (OR, 6.9; *P* < 0.001) were significant independent factors associated with prolonged operative time.

**Table 2:** Univariate and multivariable analyses of factors associated with surgical difficulty (prolonged operative time) following minimally invasive limited liver resection

		Univariate			Multivariable		
Variables		OR	95% CI	*P* value	OR	95% CI	*P* value
Age	> 75 years		0.45–2.59	0.87			
Sex	Male		0.34–1.80	0.57			
Body Mass Index	≥ 27 kg/m^2^	3.3	1.18–9.32	0.02		0.90–10.1	0.07
Repeat hepatectomy			0.67–4.76	0.24			
Liver cirrhosis	F4		0.25–2.12	0.73			
Tumor location	Posterosuperior		0.66–3.91	0.29			
Type of resection	Scooping out		0.35–1.64	0.76			
Liver transection depth	for each 1 cm	1.8	1.14–2.78	0.01	2.0	1.21–3.35	0.004
Procedure	LLR	5.3	2.29–12.2	< 0.001	6.9		< 0.001

*OR *odds ratio, *CI* confidence interval, *LLR* laparoscopic liver resection

### Subgroup analysis for outcomes stratified by the cutoff value of LTD

The receiver operating characteristic curves of LTD for predicting a prolonged operative time identified a cutoff value of 2.5 cm (area under the curve, 0.63; Fig. 3c). The surgical outcomes of limited RLR and LLR stratified by a cut-off value of 2.5 cm are shown in Table 3. In the analysis of patients with LTD ≤ 2.5 cm, no significant differences were found between the groups regarding postoperative outcomes. In the analysis of patients with LTD > 2.5 cm, the limited RLR group had a significantly shorter operative time (145 vs. 231 min, *P* < 0.001), less blood loss (nil vs. 100 mL, *P* = 0.006), and a higher rate of textbook outcomes (77% vs. 42%, *P* = 0.01) than the LLR group.

**Table 3:** The surgical outcomes of limited RLR and LLR stratified by liver transection depth

	Liver transection depth *≤* 2.5 cm			Liver transection depth > 2.5 m		
Variables	RLR (*n* = 26)	LLR (*n* = 23)	*P* value	RLR (*n* = 30)	LLR (*n* = 26)	*P* value
Liver parenchymal transection						
CUSA	9 (34.6)	23 (100)	< 0.001	18 (60.0)	26 (100)	0.001
Water jet	2 (7.7)	0 (0)		3 (10.0)	0 (0)	
Operation time, min	137 (110–178)	176 (136–218)	0.02	145 (123–178)	231 (178–267)	< 0.001
Liver transection time, min	30 (16–56)	53 (45–86)	0.002	52 (31–72)	87 (75–117)	< 0.001
Liver transection area, cm^2^	23 (16–37)	24 (20–37)	0.34	54 (43–76)	50 (39–66)	0.24
Blood loss, ml	0 (0–53)	10 (0–100)	0.13	0 (0–55)	100 (0–285)	0.006
Conversion to open surgery	0 (0)	1 (4.4)	0.28	0 (0)	3 (11.6)	0.06
Specimen weight, g	15 (10–30)	30 (20–50)	0.04	63 (45–108)	94 (40–134)	0.59
Positive surgical margin	1 (4.0)	0 (0)	0.35	1 (3.5)	3 (11.5)	0.25
Postoperative factor						
Mortality	0 (0)	0 (0)	1.000	0 (0)	0 (0)	1.000
Major complication (CDc ≥ IIIa)	1 (3.9)	0 (0)	0.34	3 (10.0)	2 (7.1)	0.76
Bile leak (≥ Grade B)	1 (3.9)	0 (0)	0.34	1 (3.3)	0 (0)	0.35
Hospital stay, day	7 (5–7)	7 (6–7)	0.32	7 (6–8)	9 (7–10)	0.21
Readmission	0 (0)	0 (0)	1.000	0 (0)	1 (3.9)	0.28
Achievement of textbook outcome	21 (80.8)	20 (87.0)	0.56	23 (76.7)	11 (42.3)	0.01

Values are reported as n (%), or median (interquartile range)

*RLR* robotic liver resection, *LLR* laparoscopic liver resection, *CUSA* Cavitron ultrasonic surgical aspirator, *CDc* Clavien–Dindo classification

## Discussion

To our knowledge, this is the first study to investigate the effects of LTD on the outcomes of patients who underwent limited RLR. The overall operative outcomes were superior in limited RLR than in limited LLR, with equivalent postoperative outcomes. Moreover, our results suggest that robotic surgery may be more effective for limited liver resection in patients with deeper LTD. We also found that LTD was an independent predictor of surgical difficulty in minimally invasive limited liver resection.

The Iwate Criteria classify the difficulty of laparoscopic liver surgery into four levels based on five preoperative factors: tumor location, extent of hepatic resection, tumor size, and proximity to major vessels [4]. Although other parameters, such as liver transection diameter, area, and resection shape, have been reported as independent indicators for estimating the complexity of LLR [6, 12, 15], these parameters have not yet been validated in RLR. Even in cases of limited RLR, the surgical difficulty differs depending on the LTD. Therefore, our study investigated the effects of LTD on patient outcomes and provides novel insights.

Our results demonstrated that increased LTD correlated with prolonged liver transection and total operative time. Interestingly, limited RLR showed approximately twice the liver transection speed of LLR, regardless of LTD. Consequently, the difference in transection time between RLR and LLR tended to increase as LTD increased. The superior efficiency of RLR can be attributed to the advantages of the robotic platform. Robotic multi-joint instruments provide stable and consistent countertraction, a critical factor when working in deep and confined transection planes, facilitating the rapid and precise use of laparoscopic CUSA [16]. This allows more efficient tissue dissection and less time spent on hemostasis during the transection phase. Given that prolonged operative time is a known factor influencing postoperative outcomes following liver surgery [17], our identification of LTD as an independent risk factor for extended operative time is clinically significant.

Furthermore, our findings revealed that LLR was associated with a high incidence of perioperative adverse events, including conversion to open surgery, major complications, positive surgical margins, and prolonged hospital stay, especially in resections deeper than 2.5 cm. This difference in adverse events was not observed in shallow liver resections, highlighting the specific benefits of robotic assistance in challenging deep dissections. Conversely, our results suggest that RLR can mitigate these disadvantages because its ability to facilitate precise vascular dissection and meticulous hemostasis plays a pivotal role in minimizing blood loss and preventing complications, particularly in complex deep transection planes. This enhanced precision may contribute to better overall postoperative outcomes, including a high rate of textbook outcomes, signifying a comprehensive measure of surgical quality and patient recovery. Therefore, our findings suggest that RLR is superior to LLR in managing deeper liver resections, effectively avoiding the many clinical disadvantages associated with laparoscopic approaches in challenging cases.

This study had several limitations, such as the limited number of patients, its single-center design, and the lack of long-term outcomes. Future studies with larger sample sizes at multiple centers should investigate the surgical difficulties associated with RLR.

## Conclusions

This study investigated the effects of LTD on surgical outcomes in patients who underwent limited RLR. Our findings indicate that LTD may be a significant factor in estimating surgical difficulty in limited RLR. Moreover, RLR could provide superior outcomes compared to LLR in cases of limited liver resection requiring deeper LTD.

## Supplementary Information

Below is the link to the electronic supplementary material.

Supplementary file 1 (PDF 171 KB)

## Data Availability

All data generated or analyzed during this study are included in this published article.
