# Supplementary material for: The impact of liver transection depth on surgical difficulty in robotic versus laparoscopic limited liver resection (TAKUMI-5)
Source: Langenbecks Arch Surg. 2025 Nov 27;411(1):22. doi: 10.1007/s00423-025-03916-0 (PMC12660394; doi:10.1007/s00423-025-03916-0)

Supplementary Information

Article title: The impact of liver transection depth on surgical difficulty in robotic versus laparoscopic limited liver resection (TAKUMI-5)

Journal name: Langenbeck's Archives of Surgery

Authors and affiliations:

Tomokazu Fuji, Kosei Takagi, Kazuya Yasui, Atene Ito, Takeyoshi Nishiyama, Yasuo Nagai, Shohei Yokoyama, and Toshiyoshi Fujiwara

Department of Gastroenterological Surgery, Okayama University Graduate School of Medicine, Dentistry, and Pharmaceutical Sciences, Okayama, Japan

Corresponding author:

Kosei Takagi, MD, PhD

E-mail: kotakagi15@gmail.com

Fig. S1 Relation between liver transection depth and (**A**) transection time and (**B**) transection speed.

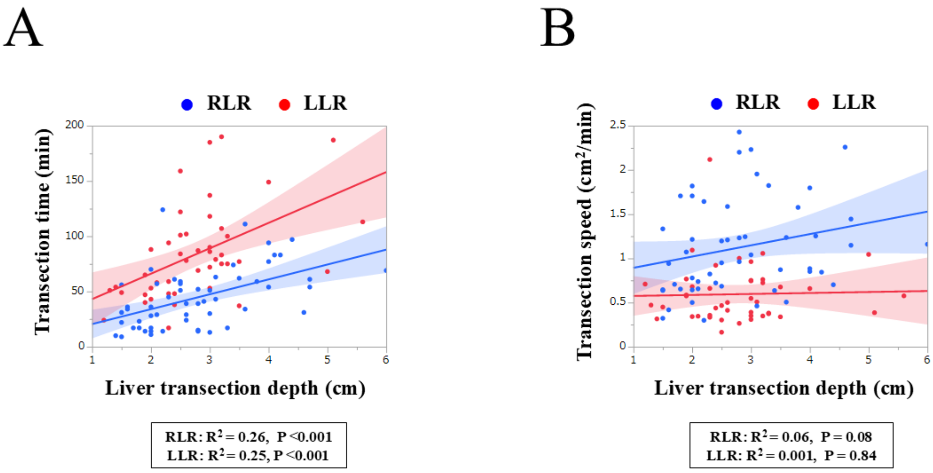

Supplement: Supplementary file 1 — (PDF 171 KB) [file 423_2025_3916_MOESM1_ESM.pdf]
